# A comparison of patient visits to osteopathic and allopathic general and family medicine physicians: results from the National Ambulatory Medical Care Survey, 2003–2004

**DOI:** 10.1186/1750-4732-1-2

**Published:** 2007-01-12

**Authors:** John C Licciardone

**Affiliations:** 1 Osteopathic Research Center, University of North Texas Health Science Center-Texas College of Osteopathic Medicine, Fort Worth, TX, USA

## Abstract

**Background:**

Osteopathic philosophy is consistent with an emphasis on primary care and suggests that osteopathic physicians may have distinctive ways of interacting with their patients.

**Methods:**

The National Ambulatory Medical Care Survey (NAMCS) was used to derive national estimates of utilization of osteopathic general and family medicine physicians during 2003 and 2004 and to examine the patient characteristics and physician-patient interactions of these osteopathic physicians. All analyses were performed using complex samples software to appropriately weigh outcomes according to the multistage probability sample design used in NAMCS and multivariate modeling was used to control for potential confounders.

**Results and discussion:**

When weighted according to the multistage probability sample design used, the 6939 patient visits studied represented an estimated 341.4 million patient visits to general and family medicine specialists in the United States, including 64.9 million (19%) visits to osteopathic physicians and 276.5 million (81%) visits to allopathic physicians. Osteopathic physicians were a major source of care in the Northeast (odds ratio [OR], 2.94; 95% confidence interval [CI], 1.42–6.08), providing more than one-third of general and family medicine patient visits in this geographic region. Pediatric and young adult patients (OR, 0.64; 95% CI, 0.45–0.91), Hispanics (OR, 0.63; 95% CI, 0.40–1.00), and non-Black racial minority groups (OR, 0.39; 95% CI, 0.18–0.82) were less likely to visit osteopathic physicians. There were no significant differences between osteopathic and allopathic physicians with regard to the time spent with patients, provision of five common preventive medicine counseling services, or a focus on preventive care during office visits.

**Conclusion:**

Osteopathic physicians are a major source of general and family medicine care in the United States, particularly in the Northeast. However, pediatric and young adult patients, Hispanics, and non-Black racial minorities underutilize osteopathic physicians. There is little evidence to support a distinctive approach to physician-patient interactions among osteopathic physicians in general and family medicine, particularly with regard to time spent with patients and preventive medicine services.

## Background

Osteopathic philosophy maintains that: (1) the body is a unit and the person is a unit of body, mind, and spirit; (2) the body is capable of self-regulation, self-healing, and health maintenance; (3) structure and function are reciprocally interrelated; and (4) rational treatment is based on an understanding of the basic principles of body unity, self-regulation, and the interrelationship of structure and function [1]. An emphasis on the provision of primary care services, particularly in general and family medicine, is a natural contemporary outgrowth of osteopathic philosophy. The American Osteopathic Association (AOA) estimates that of the 40,067 osteopathic physicians (DOs) in active practice (excluding those in postgraduate and other postdoctoral training programs), 18,765 (47%) are in the specialty of general and family medicine, 3278 (8%) are in internal medicine, and 1663 (4%) are in pediatrics [2]. Trends in graduate medical education also show a rise in osteopathic trainees entering family medicine residency programs accredited by the Accreditation Council for Graduate Medical Education (ACGME) [3], and suggest that the primary care medical workforce of the future will include more osteopathic physicians [4].

Some claim that osteopathic physicians, because of their philosophy and orientation toward primary care, may interact with their patients in ways that are distinctive from other health care providers. This has been characterized as a holistic or patient-centered approach, with a focus on preventive care [5]. In the Maine Osteopathic Outcomes Study (MOOS), a 26-item index of physician-patient communications hypothesized to be reflective of osteopathic philosophy was constructed [6]. The communications and patient interactions of osteopathic physicians were then compared with those of allopathic physicians (MDs) by using audiotapes. The MOOS findings demonstrated that osteopathic physicians did indeed score higher than allopathic physicians on many of the items. Specifically, osteopathic physicians were more likely than allopathic physicians to discuss preventive measures specific to the presenting complaint, health issues in relation to family life and social activities, and the patient's emotional state.

The First Osteopathic Survey of Health Care in America (OSTEOSURV-I), a random national telephone survey, addressed patient satisfaction with various elements of health care provided by osteopathic physicians, allopathic physicians, chiropractors, and other non-physician clinicians [7]. In OSTEOSURV-I, patients of osteopathic physicians tended to report the greatest levels of satisfaction on such items as emphasis on wellness, use of educational materials, and time spent with the health care provider. Subsequently, the Second Osteopathic Survey of Health Care in America (OSTEOSURV-II) also identified factors associated with the use of an osteopathic physician as the respondent's main health care provider [8]. In OSTEOSURV-II, women were more likely than men to use osteopathic physicians, whereas racial or ethnic minority groups were less likely to use osteopathic physicians.

Investigators at Michigan State University College of Osteopathic Medicine (MSUCOM) conducted a random national mail survey of osteopathic physicians to identify philosophical and practical differences that they believed distinguished them from their allopathic counterparts [9]. Osteopathic physicians reported holistic medicine as their most distinguishing philosophical characteristic, and use of osteopathic manipulative treatment (OMT), a caring physician-patient relationship, and a "hands-on" style as the most common practical differences from allopathic physicians. Other smaller and older surveys of the general population in various municipalities generally supported the interpersonal manner and communication skills of osteopathic physicians [10,11].

While these cited studies provide some insight into osteopathic medicine and the physician-patient interactions characteristic of osteopathic primary care, they are limited in various ways. The MOOS study involved only 18 physicians and 54 patients in a geographically limited area; therefore, its findings may not be generalizable on a national level [6]. Although the OSTEOSURV studies were random national surveys based on a validated survey instrument [12], the numbers of respondents (1106 on OSTEOSURV-I [7] and 499 on OSTEOSURV-II) [8] were not sufficiently large to ensure adequate statistical power in testing certain hypotheses, particularly when subgroup analyses were performed. The MSUCOM survey was limited to only osteopathic physicians, and its low response rate (979 respondents out of 2946 eligible contacts) may have been indicative of a self-selection bias [9]. The present study was performed to extend our knowledge of osteopathic medicine by deriving national estimates of the use of osteopathic general and family medicine physicians and examining the patient characteristics and physician-patient interactions of these osteopathic physicians.

## Methods

### Overview of National Ambulatory Medical Care Survey design

The concept of the National Ambulatory Medical Care Survey (NAMCS) to collect data on medical care provided in physician offices in the United States was developed over 30 years ago [13]. Documentation of the NAMCS instrument, methodology, and data files that served as the basis for this study is available elsewhere [14,15]. The NAMCS contains data on 25,288 patient visits to 1114 physician offices during the 2003 calendar year and 25,286 patient visits to 1121 physician offices during the 2004 calendar year. These patient visits were selected using a multistage probability sample design, with primary sampling units (PSUs) selected in the first stage, physician practices within PSUs in the second stage, and patient visits to the selected physicians in the third stage.

The first stage included 112 PSUs, which consisted of counties, groups of counties, county equivalents (e.g. parishes), towns, townships, minor civil divisions, or metropolitan statistical areas (MSAs). These PSUs comprised a probability subsample of those used in the 1985–1994 National Health Interview Surveys [16]. The latter, which covered all 50 states and the District of Columbia, were stratified by demographic and socioeconomic variables and then selected with probability proportional to their size. Stratification was done within four geographic regions by MSA and non-MSA status.

The second stage consisted of a probability sample of practicing physicians selected from the master files of the AOA and the American Medical Association (AMA). Within each PSU, all eligible physicians were stratified by specialty: general and family practice, internal medicine, pediatrics, general surgery, obstetrics and gynecology, orthopedic surgery, cardiovascular diseases, dermatology, urology, psychiatry, neurology, ophthalmology, otolaryngology, and all other specialties. A separate specialty designation was provided for osteopathic physicians.

The third stage involved selection of patient visits within the practices of participating physicians. Initially, the physician was randomly assigned to one of the 52 weeks within the calendar year. Then, a systematic random sample of patient visits was selected by the physician during the assigned week. The sampling rate of patient visits varied from a 20% sample for very large practices to 100% for very small practices as determined by a presurvey interview [17]. In this manner, data from about 30 patient visits were recorded by each physician during the assigned week.

### The NAMCS sampling frame and sample size

The sampling frame for NAMCS included all physicians in the master files of the AOA and AMA prior to the start of the survey year who met the following criteria: (1) office-based; (2) principally engaged in patient care activities; (3) nonfederally employed; and (4) not in the specialties of anesthesiology, pathology, or radiology. During 2003 and 2004, a total of 6000 physicians, including 460 (8%) osteopathic physicians and 5540 (92%) allopathic physicians, were initially screened. Of these, 2032 (34%) did not meet the four criteria listed above and were ineligible. The most common reasons for being ineligible were that the physician was retired, deceased, or employed in teaching, research, or administration. Of the remaining 3968 eligible physicians, 2779 (70%) participated in NAMCS. Among the participating physicians, 544 (20%) saw no patients during their assigned reporting period because of vacations, illness, or other reasons for being temporarily not in practice.

### Physician-patient encounters

The basic sampling unit for NAMCS is the physician-patient encounter or "visit." The following types of contacts were excluded: telephone calls, visits outside the physician's office (e.g., house calls), visits made in hospital settings (unless the physician had a private office in a hospital), visits made in institutional settings that had primary responsibility for the patient's care (e.g., nursing homes), and visits to the physician's office for administrative purposes only (e.g., to leave a specimen, pay a bill, or pick up insurance forms).

### The NAMCS data collection and processing

Data for NAMCS were collected by the physician with assistance from office staff when possible. Patient record forms were used to collect the data for each selected visit. The NAMCS field staff performed completeness checks of the patient record forms prior to submission for central data processing. Detailed editing instructions were provided to reclassify or recode ambiguous or inconsistent data entries. Quality control measures, which were used to verify the accuracy of computer data entry, indicated that the mean keying error rate was 0.1% for nonmedical items and that discrepancy rates ranged from 0.0% to 1.1% for required medical items.

Item nonresponse rates were 5% or less for most variables. Major exceptions (nonresponse rate) included: ethnicity (20%), race (18%), tobacco use (30%), and time spent with physician (16%). Missing data for birth year (4%), sex (4%), race (18%), ethnicity (20%), and time spent with physician (16%) were imputed by assigning the value from a randomly selected patient record form representing another patient with similar known characteristics. Such imputations were performed according to physician specialty, geographic region (state was used instead of geographic region to impute ethnicity), and primary diagnosis codes.

### Patient visit weight

Each record in the NAMCS data file was assigned a patient visit weight based on four factors: (1) probability of being selected by the three-stage sampling design; (2) adjustment for nonresponse; (3) adjustment for physician specialty group; and (4) weight smoothing to minimize the impact of a few physician outliers whose final visit weights were large relative to those for the remaining physicians. Thus, by applying the relevant patient visit weights to each of the 50,574 records in the 2003 and 2004 NAMCS data files, an estimated 1.82 billion physician office visits in the United States were represented. When weighted according to the multistage probability sample design, the NAMCS data may be used to derive unbiased national estimates of ambulatory medical care services utilization and to further characterize such services.

### Data management and analyses

The electronic files containing the 2003 and 2004 NAMCS data were acquired from the National Center for Health Statistics. The files were merged and analyzed using SPSS Version 14.0 for Windows (SPSS Inc., Chicago, IL). Because the multistage probability design of NAMCS includes clustering, stratification, and the assignment of unequal probabilities of selection to sample units, all analyses were performed with the SPSS complex samples module to accurately compute estimates of population parameters and their standard errors [18]. The present study is based only on patient visits to physician offices for general or family medicine. Patient visits were excluded from analysis if a physician was not seen during the visit or if the physician seen was not the patient's primary care physician.

The primary variables of interest included patient characteristics, place of residence, episode of care (initial or follow-up visit), major reason for the physician office visit (acute problem, chronic problem, preventive care, or other reason), length of time spent with patient, and five common patient counseling services (diet or nutrition counseling, weight reduction counseling, exercise counseling, tobacco use or exposure counseling, and mental health or stress reduction counseling). The time spent with patients and provision of each of these five types of patient counseling was used to assess physician-patient interactions.

National estimates of the use of osteopathic and allopathic physicians in the specialty of general and family medicine were derived. Multiple logistic regression was used to compute adjusted odds ratios (ORs) and 95% confidence intervals (CIs) for the use of osteopathic physicians, controlling for potential confounders, including patient characteristics, place of residence, episode of care, and major reason for the physician office visit. Additionally, the osteopathic population attributable percentage (PAP) was used to express the percentage of national office visits provided by osteopathic physicians.

The time spent with patients by osteopathic and allopathic physicians was compared using a multiple linear regression model that controlled for the potential confounders delineated above. This model was used to compute adjusted means and standard errors of time spent with patients according to physician provider type and levels of each potential confounder. National estimates of the provision of diet or nutrition counseling, weight reduction counseling, exercise counseling, tobacco use or exposure counseling, and mental health or stress reduction counseling were also derived. The provision of these counseling services by osteopathic and allopathic physicians was compared using multiple logistic regression to compute adjusted ORs and 95% CIs that controlled for the potential confounders listed above. All hypotheses were tested at the .05 level of statistical significance.

## Results

### National utilization estimates

There were an estimated 341.4 million patient visits to general and family medicine specialists during 2003 and 2004, including 64.9 million (19%) visits to osteopathic physicians and 276.5 million (81%) visits to allopathic physicians. The patient and visit characteristics according to physician provider type are presented in Table 1. Patients in the Northeast were more likely to visit osteopathic physicians than patients in the West (OR, 2.94; 95% CI, 1.42–6.08). In the Northeast, more than one-third of the general and family medicine patient visits (18.0 million of 52.1 million visits) were provided by osteopathic physicians. Patients who were 24 years of age or younger were significantly less likely to visit osteopathic physicians than patients who were 65 years of age or older (OR, 0.64; 95% CI, 0.45–0.91). Also, Hispanics (OR, 0.63; 95% CI, 0.40–1.00) and non-Black racial minority groups (OR, 0.39; 95% CI, 0.18–0.82) were less likely to visit osteopathic physicians than non-Hispanics or Whites, respectively.

**Table 1:** National estimates of general and family medicine patient visits (in millions) according to physician provider type.*

	**Physician Provider Type**								
						
	**DO**		**MD**						
							
**Patient or Visit Characteristic**	**No.**	**%**	**No.**	**%**	**OR†**	**95% CI**			**Osteopathic PAP‡**
**Age, yr**									
≤24	9.0	13.9	57.2	20.7	0.64	0.45	-	0.91	14
25–44	15.4	23.7	67.9	24.5	0.80	0.60	-	1.06	18
45–64	23.4	36.1	90.7	32.8	0.95	0.75	-	1.20	21
≥65	17.1	26.3	60.8	22.0	1§		...		22
**Sex**									
Female	38.4	59.2	161.2	58.3	1§		...		19
Male	26.5	40.8	115.3	41.7	0.99	0.85	-	1.14	19
**Race**									
White	59.3	91.4	236.9	85.7	1§		...		20
Black	4.6	7.1	28.7	10.4	0.69	0.38	-	1.27	14
Other	0.9	1.5	10.9	4.0	0.39	0.18	-	0.82	8
**Ethnicity**									
Non-Hispanic	61.2	94.3	246.4	89.1	1§		...		20
Hispanic	3.7	5.7	30.1	10.9	0.63	0.40	-	1.00	11
**Geographic region**									
Northeast	18.0	27.8	34.1	12.3	2.94	1.42	-	6.08	35
Midwest	20.9	32.2	73.3	26.5	1.79	0.93	-	3.43	22
South	14.6	22.4	105.9	38.3	0.83	0.41	-	1.67	12
West	11.4	17.6	63.1	22.8	1§		...		15
**Residence in MSA**									
Yes	57.4	88.5	220.4	79.7	1§		...		21
No	7.4	11.5	56.1	20.3	0.43	0.18	-	1.06	12
**Episode of care**									
Initial visit	23.6	36.3	108.1	39.1	1§		...		18
Follow-up visit	33.0	50.8	113.1	40.9	1.27	0.97	-	1.67	23
Unknown	8.3	12.9	55.3	20.0	0.74	0.38	-	1.46	13
**Major reason for visit**									
Acute problem	30.2	46.5	126.5	45.7	1§		...		19
Chronic problem	27.3	42.0	98.0	35.4	0.92	0.69	-	1.22	22
Preventive care	6.3	9.7	42.7	15.4	0.92	0.45	-	1.86	13
Other/unknown	1.2	1.8	9.3	3.4	0.53	0.27	-	1.04	11

*Derived from the 2003 and 2004 National Ambulatory Medical Care Survey using population estimates based on patient visit weights. CI denotes confidence interval; DO, osteopathic physician; MD, allopathic physician; MSA, metropolitan statistical area; OR, odds ratio, PAP, population attributable percentage.

†ORs are for the utilization of osteopathic physicians relative to allopathic physicians, and are adjusted for the other patient or visit characteristics in the table.

‡Osteopathic PAP refers to the percentage of physician visits provided by osteopathic physicians.

§Reference category for OR.

### Time spent with physician

The adjusted national estimates of time spent with physician during general and family medicine patient visits are presented in Table 2. Patients who were 24 years of age or younger spent significantly less time (± SE) with physicians than patients who were 65 years of age or older (17.21 ± 0.78 min vs 20.36 ± 0.82 min; P < .001). Patients spent significantly more time with physicians during visits for chronic problems (18.43 ± 0.75 min; P=.01) and preventive care (22.52 ± 1.22 min; P < .001) than during visits for acute problems (17.16 ± 0.80 min). Neither the crude nor adjusted times spent with osteopathic physicians were significantly different than times spent with allopathic physicians.

**Table 2:** National estimates of time spent with physician during general and family medicine patient visits.*

	**Time† (min)**		
		
**Patient or Visit Characteristic**	**Mean**	**SE**	**P**
**Age, yr**			
≤24	17.21	0.78	< .001
25–44	20.15	0.91	.68
45–64	20.27	0.91	.83
≥65	20.36	0.82	‡
**Sex**			
Female	19.50	0.84	‡
Male	19.50	0.82	.91
**Race**			
White	19.55	0.62	‡
Black	19.23	0.90	.66
Other	19.71	1.47	.90
**Ethnicity**			
Non-Hispanic	19.42	0.75	‡
Hispanic	19.57	0.98	.80
**Geographic region**			
Northeast	20.90	1.21	.21
Midwest	18.23	0.78	.09
South	19.26	0.97	.66
West	19.61	0.90	‡
**Residence in MSA**			
Yes	19.16	0.81	‡
No	19.84	0.99	.38
**Episode of care**			
Initial visit	19.36	0.93	‡
Follow-up visit	19.35	0.94	.99
Unknown	19.78	0.92	.65
**Major reason for visit**			
Acute problem	17.16	0.80	‡
Chronic problem	18.43	0.75	.01
Preventive care	22.52	1.22	< .001
Other/unknown	19.88	1.39	.02
**Provider type**			
Allopathic physician	19.65	0.77	‡
Osteopathic physician	19.34	1.04	.71

*Derived from the 2003 and 2004 National Ambulatory Medical Care Survey using population estimates based on patient visit weights. MSA denotes metropolitan statistical area.

†Time spent with physician is adjusted for the patient or visit characteristics in the table.

‡Reference category for contrasts.

### Patient counseling

The national estimates of patient counseling during the 341.4 million general and family medicine patient visits studied were as follows: diet or nutrition counseling, 65.4 million (19%); weight reduction counseling, 17.0 million (5%); exercise counseling, 45.7 million (13%); tobacco use or exposure counseling, 13.7 million (4%); and mental health or stress reduction counseling, 19.6 million (6%).

The patient and visit characteristics associated with the provision of diet or nutrition counseling, weight reduction counseling, and exercise counseling are presented in Tables 3, 4, 5, respectively. The factors associated with such counseling were generally similar across these analyses. Each of these three types of patient counseling was provided significantly more often during visits for chronic problems, preventive care, and other or unknown reasons than during visits for acute problems. Patients who were 24 years of age or younger were significantly less likely, and patients who were 25 to 44 years of age were significantly more likely, to receive these three types of counseling than patients who were 65 years of age or older. Non-Black racial minority groups were more likely to receive such counseling than Whites.

**Table 3:** National estimates of diet or nutrition counseling during general and family medicine patient visits (in millions).*

	**Counseling Provided**							
					
	**Yes**		**No**					
						
**Patient or Visit Characteristic**	**No.**	**%**	**No.**	**%**	**OR† **	**95% CI**		
**Age, yr**								
≤24	8.6	13.1	57.6	20.9	0.68	0.49	-	0.94
25–44	13.8	21.1	69.5	25.2	0.92	0.73	-	1.17
45–64	27.1	41.5	87.0	31.5	1.23	1.02	-	1.49
≥65	15.9	24.4	61.9	22.4	1‡		...	
**Sex**								
Female	36.5	55.8	163.1	59.1	1‡		...	
Male	28.9	44.2	112.9	40.9	1.14	0.99	-	1.31
**Race**								
White	53.8	82.3	242.4	87.8	1‡		...	
Black	7.9	12.0	25.4	9.2	1.35	0.94	-	1.93
Other	3.7	5.7	8.2	3.0	1.71	1.16	-	2.52
**Ethnicity**								
Non-Hispanic	55.6	85.1	252.0	91.3	1‡		...	
Hispanic	9.8	14.9	24.0	8.7	1.79	1.15	-	2.80
**Geographic region**								
Northeast	9.1	13.8	43.1	15.6	0.96	0.56	-	1.64
Midwest	15.2	23.2	79.0	28.6	0.84	0.54	-	1.29
South	25.0	38.2	95.5	34.6	1.10	0.68	-	1.78
West	16.1	24.7	58.4	21.1	1‡		...	
**Residence in MSA**								
Yes	54.4	83.3	223.3	80.9	1‡		**... **	
No	10.9	16.7	52.6	19.1	0.92	0.70	-	1.19
**Episode of care**								
Initial visit	13.5	20.6	118.1	42.8	1‡		**... **	
Follow-up visit	35.6	54.5	110.4	40.0	1.78	1.43		2.22
Unknown	16.2	24.8	47.5	17.2	1.29	0.82		2.01
**Major reason for visit**								
Acute problem	16.8	25.7	140.0	50.7	1‡			
Chronic problem	32.1	49.1	93.1	33.7	1.94	1.53	-	2.47
Preventive care	13.8	21.1	35.2	12.8	3.04	1.92	-	4.81
Other/unknown	2.7	4.1	7.8	2.8	2.10	1.01	-	4.34
**Provider type**								
Allopathic physician	55.0	84.2	221.4	80.2	1‡			
Osteopathic physician	10.3	15.8	54.7	19.8	0.79	0.50	-	1.25

*Derived from the 2003 and 2004 National Ambulatory Medical Care Survey using population estimates based on patient visit weights. CI denotes confidence interval; MSA, metropolitan statistical area; OR, odds ratio.

†ORs are for provision of diet or nutrition counseling, and are adjusted for the other patient or visit characteristics in the table.

‡Reference category for OR.

**Table 4:** National estimates of weight reduction counseling during general and family medicine patient visits (in millions).*

	**Counseling Provided**							
					
	**Yes**		**No**					
						
**Patient or Visit Characteristic**	**No.**	**%**	**No.**	**%**	**OR† **	**95% CI**		
**Age, yr**								
≤24	0.9	5.2	65.3	20.1	0.34	0.16	-	0.71
25–44	4.1	24.3	79.1	24.4	1.35	0.91	-	2.02
45–64	8.5	49.7	105.8	32.6	1.75	1.29	-	2.38
≥65	3.5	20.8	74.3	22.9	1‡		...	
**Sex**								
Female	10.2	60.2	189.5	58.4	1‡		...	
Male	6.8	39.8	135.0	41.6	0.90	0.70	-	1.17
**Race**								
White	12.7	74.9	283.5	87.4	1‡		...	
Black	3.2	18.8	30.1	9.3	2.20	1.46	-	3.30
Other	1.1	6.3	10.8	3.3	2.18	1.26	-	3.77
**Ethnicity**								
Non-Hispanic	15.2	89.6	292.3	90.1	1‡		...	
Hispanic	1.8	10.4	32.0	9.9	1.10	0.65	-	1.89
**Geographic region**								
Northeast	3.2	19.0	48.9	15.1	1.52	0.83	-	2.79
Midwest	3.1	18.0	91.2	28.1	0.73	0.40	-	1.34
South	7.2	42.2	113.2	34.9	1.29	0.71	-	2.36
West	3.5	20.8	71.0	21.9	1‡		...	
**Residence in MSA**								
Yes	14.0	82.0	264.1	81.4	1‡		...	
No	3.1	18.0	60.5	18.6	1.06	0.63	-	1.80
**Episode of care**								
Initial visit	3.4	20.3	128.1	39.5	1‡		...	
Follow-up visit	9.6	56.0	136.6	42.1	1.30	0.92	-	1.83
Unknown	4.0	23.7	59.6	18.4	0.61	0.29	-	1.28
**Major reason for visit**								
Acute problem	3.7	21.5	153.1	47.2	1‡		...	
Chronic problem	9.0	53.2	116.1	35.8	2.47	1.75	-	3.49
Preventive care	3.7	21.5	45.4	14.0	6.39	2.91	-	14.05
Other/unknown	0.6	3.8	9.9	3.0	2.73	1.19	-	6.27
**Provider type**								
Allopathic physician	13.9	81.8	262.8	81.0	1‡		...	
Osteopathic physician	3.1	18.2	61.8	19.0	0.96	0.58	-	1.58

*Derived from the 2003 and 2004 National Ambulatory Medical Care Survey using population estimates based on patient visit weights. CI denotes confidence interval; MSA, metropolitan statistical area; OR, odds ratio.

†ORs are for provision of weight reduction counseling, and are adjusted for the other patient or visit characteristics in the table.

‡Reference category for OR.

**Table 5:** National estimates of exercise counseling during general and family medicine patient visits (in millions).*

	**Counseling Provided**							
					
	**Yes**		**No**					
						
**Patient or Visit Characteristic**	**No.**	**%**	**No.**	**%**	**OR† **	**95% CI**		
**Age, yr**								
≤24	3.8	8.4	62.4	21.1	0.47	0.32	-	0.69
25–44	11.1	24.2	72.2	24.4	1.22	0.93	-	1.61
45–64	20.9	45.6	93.3	31.5	1.54	1.24	-	1.90
≥65	10.0	21.8	67.9	23.0	1‡		...	
**Sex**								
Female	25.5	55.7	174.1	58.9	1‡		...	
Male	20.3	44.3	121.5	41.1	1.14	0.98	-	1.33
**Race**								
White	38.2	83.5	258.1	87.3	1‡		...	
Black	4.9	10.7	28.4	9.6	1.17	0.81	-	1.69
Other	2.7	5.9	9.2	3.1	1.56	1.04	-	2.33
**Ethnicity**								
Non-Hispanic	39.4	86.2	268.2	90.7	1‡		...	
Hispanic	6.3	13.8	27.5	9.3	1.40	0.83	-	2.38
**Geographic region**								
Northeast	6.7	14.7	45.5	15.4	0.84	0.46	-	1.52
Midwest	10.5	23.0	83.7	28.3	0.70	0.43	-	1.15
South	15.6	34.2	105.0	35.5	0.81	0.44	-	1.46
West	12.9	28.1	61.6	20.8	1‡		...	
**Residence in MSA**								
Yes	38.9	85.0	238.9	80.8	1‡		...	
No	6.9	15.0	56.7	19.2	0.86	0.59	-	1.24
**Episode of care**								
Initial visit	9.9	21.6	121.8	41.2	1‡		...	
Follow-up visit	24.7	53.9	121.5	41.1	1.65	1.24	-	2.21
Unknown	11.2	24.5	52.5	17.7	1.34	0.81	-	2.24
**Major reason for visit**								
Acute problem	12.4	27.1	144.3	48.8	1‡		...	
Chronic problem	21.7	47.5	103.5	35.0	1.65	1.20	-	2.27
Preventive care	9.3	20.3	39.7	13.4	2.50	1.58	-	3.97
Other/unknown	2.3	5.1	8.2	2.8	2.42	1.08	-	5.41
**Provider type**								
Allopathic physician	37.8	82.6	238.6	80.7	1‡		...	
Osteopathic physician	7.9	17.4	56.9	19.3	0.88	0.52	-	1.50

*Derived from the 2003 and 2004 National Ambulatory Medical Care Survey using population estimates based on patient visit weights. CI denotes confidence interval; MSA, metropolitan statistical area; OR, odds ratio.

†ORs are for provision of exercise counseling, and are adjusted for the other patient or visit characteristics in the table.

‡Reference category for OR.

The patient and visit characteristics associated with the provision of tobacco use or exposure counseling, and mental health or stress reduction counseling, are presented in Tables 6 and 7, respectively. Patients who were 25 to 44 years of age and patients who were 45 to 64 years of age were significantly more likely to receive these two types of counseling than patients who were 65 years of age or older. There were no significant differences between osteopathic and allopathic physicians with regard to the provision of any of the five patient counseling services studied.

**Table 6:** National estimates of tobacco use or exposure counseling during general and family medicine patient visits (in millions).*

	**Counseling Provided**							
					
	**Yes**		**No**					
						
**Patient or Visit Characteristic**	**No.**	**%**	**No.**	**%**	**OR† **	**95% CI**		
**Age, yr**								
≤24	1.3	9.8	64.9	19.8	0.85	0.45	-	1.59
25–44	4.2	30.4	79.1	24.1	2.31	1.40	-	3.82
45–64	6.4	46.3	107.8	32.9	2.44	1.51	-	3.93
≥65	1.9	13.5	76.0	23.2	1‡		...	
**Sex**								
Female	7.0	50.8	192.7	58.8	1‡		...	
Male	6.8	49.2	135.0	41.2	1.39	1.08	-	1.79
**Race**								
White	11.8	86.2	284.4	86.8	1‡		...	
Black	1.6	11.7	31.7	9.7	1.07	0.56	-	2.03
Other	0.3	2.1	11.6	3.5	0.71	0.37	-	1.37
**Ethnicity**								
Non-Hispanic	13.1	95.7	294.6	89.9	1‡		...	
Hispanic	0.6	4.3	33.2	10.1	0.44	0.21	-	0.93
**Geographic region**								
Northeast	2.1	15.4	50.1	15.3	1.23	0.64	-	2.36
Midwest	4.3	31.1	90.0	27.5	1.37	0.83	-	2.27
South	5.3	38.7	115.3	35.2	1.43	0.76	-	2.69
West	2.0	14.8	72.5	22.1	1‡		...	
**Residence in MSA**								
Yes	10.4	76.1	267.4	81.6	1‡		...	
No	3.3	23.9	60.2	18.4	1.34	0.78	-	2.31
**Episode of care**								
Initial visit	4.4	31.8	127.1	38.8	1‡		...	
Follow-up visit	5.9	42.7	140.2	42.8	1.15	0.76	-	1.73
Unknown	3.5	25.6	60.2	18.4	2.49	1.39	-	4.45
**Major reason for visit**								
Acute problem	5.7	41.4	151.1	46.1	1‡		...	
Chronic problem	5.3	38.8	119.9	36.6	1.07	0.73	-	1.57
Preventive care	2.5	17.9	46.6	14.2	0.65	0.36	-	1.19
Other/unknown	0.3	1.9	10.3	3.1	0.56	0.19	-	1.63
**Provider type**								
Allopathic physician	10.9	79.8	265.4	81.0	1‡		...	
Osteopathic physician	2.8	20.2	62.1	19.0	1.09	0.63	-	1.88

*Derived from the 2003 and 2004 National Ambulatory Medical Care Survey using population estimates based on patient visit weights. CI denotes confidence interval; MSA, metropolitan statistical area; OR, odds ratio.

†ORs are for provision of tobacco use or exposure counseling, and are adjusted for the other patient or visit characteristics in the table.

‡Reference category for OR.

**Table 7:** National estimates of mental health or stress reduction counseling during general and family medicine patient visits (in millions).*

	**Counseling Provided**							
					
	**Yes**		**No**					
						
**Patient or Visit Characteristic**	**No.**	**%**	**No.**	**%**	**OR† **	**95% CI**		
**Age, yr**								
≤24	3.1	15.7	63.1	19.6	1.88	1.21	-	2.92
25–44	6.9	35.4	76.3	23.7	3.16	2.26	-	4.43
45–64	6.9	35.1	107.2	33.3	1.95	1.38	-	2.75
≥65	2.7	13.8	75.2	23.4	1‡		**... **	
**Sex**								
Female	12.4	63.4	187.3	58.2	1‡		...	
Male	7.1	36.6	134.5	41.8	0.81	0.64	-	1.04
**Race**								
White	16.8	85.9	279.4	86.8	1‡		...	
Black	2.0	10.0	31.3	9.7	0.98	0.59	-	1.62
Other	0.8	4.1	11.1	3.4	1.23	0.65	-	2.33
**Ethnicity**								
Non-Hispanic	17.9	91.5	289.7	90.0	1‡		...	
Hispanic	1.7	8.5	32.1	10.0	0.83	0.46	-	1.52
**Geographic region**								
Northeast	4.4	22.3	47.8	14.9	1.61	0.83	-	3.13
Midwest	4.8	24.6	89.4	27.8	0.91	0.46	-	1.78
South	6.3	32.2	114.3	35.5	0.98	0.55	-	1.74
West	4.1	20.9	70.4	21.9	1‡		...	
**Residence in MSA**								
Yes	16.2	82.8	261.7	81.3	1‡		...	
No	3.4	17.2	60.2	18.7	0.95	0.64	-	1.39
**Episode of care**								
Initial visit	5.0	25.7	126.5	39.3	1‡		...	
Follow-up visit	10.7	54.8	135.5	42.1	2.11	1.33	-	3.34
Unknown	3.8	19.5	59.9	18.6	2.68	1.73	-	4.16
**Major reason for visit**								
Acute problem	7.6	39.1	149.0	46.3	1‡		...	
Chronic problem	9.1	46.3	116.2	36.1	1.17	0.76	-	1.78
Preventive care	2.5	12.8	46.5	14.4	0.53	0.31	-	0.92
Other/unknown	0.3	1.8	10.2	3.2	0.51	0.16	-	1.62
**Provider type**								
Allopathic physician	16.2	83.1	260.4	80.9	1‡		...	
Osteopathic physician	3.3	16.9	61.6	19.1	0.77	0.43	-	1.36

*Derived from the 2003 and 2004 National Ambulatory Medical Care Survey using population estimates based on patient visit weights. CI denotes confidence interval; MSA, metropolitan statistical area; OR, odds ratio.

†ORs are for provision of mental  health or stress reduction counseling, and are adjusted for the other patient or visit characteristics in the table.

‡Reference category for OR.

## Discussion

The results of this study involving a large nationally representative sample of office visits provides a clearer picture of the characteristics of patients visiting osteopathic physicians in general and family medicine, and of the physician-patient interactions occurring during such visits. Overall, there were an estimated 64.9 million ambulatory visits to osteopathic physicians in general and family medicine during 2003 and 2004. Osteopathic physicians accounted for about one-fifth of general and family medicine visits in the United States during this period.

Osteopathic medicine was founded in and has traditionally been most strongly associated with the Midwest [19]. The findings of this study, however, bring to light the disproportionately large contribution of osteopathic physicians to general and family medicine in the Northeast. Even after adjusting for potential confounders, patients in the Northeast were about three times more likely to visit an osteopathic physician for general and family medicine than patients in the West. In the Northeast, over one-third of general and family medicine patient visits were provided by osteopathic physicians. This finding can be attributed to the relative overabundance of general and family medicine visits provided by osteopathic physicians in the Northeast (28% of all osteopathic visits nationally) coupled with the relative lack of such visits provided by allopathic physicians (12% of all allopathic visits nationally).

This study also extends previous findings with regard to use of osteopathic physicians in racial or ethnic minority groups [8] by identifying Hispanics and non-Black minorities as the groups with decreased utilization of osteopathic physicians. While the reasons for decreased use of osteopathic physicians by Hispanics are not entirely clear, it is possible that the small percentage of Hispanics within the osteopathic profession may be partially responsible. Currently, only 420 (4%) of the 11,857 students enrolled in colleges of osteopathic medicine are Hispanic [2].

This study found little evidence to support a distinctive approach to physician-patient interactions among osteopathic physicians in general and family medicine. Patients spent comparable amounts of time with osteopathic and allopathic physicians during office visits, even after controlling for patient characteristics, place of residence, episode of care, and reason for the visit. There were no significant differences between osteopathic and allopathic physicians with regard to the provision of five common types of counseling within the realm of preventive medicine. Finally, there was no evidence that patients visited osteopathic physicians for preventive care more often than they visited allopathic physicians for such care.

Certainly, there are other elements of the physician-patient interaction that were not captured with the NAMCS patient record form used during office visits. However, the primary variables studied herein – time spent with patients, provision of common preventive medicine counseling services, and a focus on preventive care during office visits – represent aspects of medical care commonly emphasized as manifestations of the osteopathic philosophy. Failure to identify distinctive patterns of care rendered by osteopathic general and family medicine physicians in any of these primary variables brings into question the existence of a unique osteopathic approach to medical care. Further, although not directly measured in NAMCS, the use of OMT during office visits may have been infrequent based on the comparable amount of time spent with patients by osteopathic and allopathic physicians. One obvious factor that may have impacted the study results, attenuating differences between osteopathic and allopathic physicians, is the training of osteopathic physicians in ACGME-accredited residency programs. As such training is generally on the rise [3,4,20], its influence on osteopathic philosophy and physician-patient interactions is likely to grow.

There are several potential limitations of this study. First, with regard to information, NAMCS data were collected by the physician providers with assistance from their office staff rather than by independent survey personnel. Thus, there exists the potential for variability in the data collection process across physician providers, potentially leading to imprecision or information bias in the reported data. For example, a very brief mention of nutrition and a much more comprehensive counseling session on diet both may have met the criterion of diet or nutrition counseling during a patient visit. However, the relatively low percentages of patient visits during which the various types of counseling were reported to have occurred suggest that physician overreporting was not an important source of bias.

Second, with regard to provision of patient counseling, the implicit assumptions were that patients of osteopathic and allopathic physicians were comparable in their need for counseling and that more frequent counseling was indicative of more optimal physician-patient interactions. However, in 2003 and 2004, NAMCS did not routinely collect patient data to adequately assess the need for most of the counseling services studied and to thereby verify these assumptions. Beginning in 2005, the NAMCS patient record form routinely collects data on patient height and weight, thereby enabling future investigators to more adequately control for the need of counseling in such areas as diet or nutrition, weight reduction, and exercise.

Third, there were substantial amounts of missing data for ethnicity, race, and time spent with physician. Although these missing values were imputed by NAMCS personnel using accepted statistical methods, the imputed data cannot be validated with absolute certainty.

Finally, the selection of physicians and their patients should also be addressed. Overall, 70% of eligible physicians elected to participate in the survey. Among participating physicians, one-fifth saw no patients during their assigned reporting period for various reasons. Thus, some degree of selection bias in NAMCS cannot be ruled out. Further, the findings of this study apply only to general and family medicine physicians and cannot be extrapolated to other specialty physicians, including those in other primary care specialties.

## Conclusion

Osteopathic physicians are a major source of general and family medicine care in the United States, particularly in the Northeast. However, pediatric and young adult patients, Hispanics, and non-Black racial minorities underutilize osteopathic physicians across the nation. There is little evidence to support a distinctive approach to physician-patient interactions among osteopathic physicians in general and family medicine, particularly with regard to time spent with patients and preventive medicine services.

## Competing interests

JCL is Editor-in-Chief of *Osteopathic Medicine and Primary Care*. He was not involved in the review of the manuscript or in the editorial decision with regard to its suitability for publication.
